# *QuickStats*: Average Number of Deaths from Motor Vehicle Injuries, Suicide, and Homicide,* by Day of the Week — National Vital Statistics System, United States, 2015

**DOI:** 10.15585/mmwr.mm6622a5

**Published:** 2017-06-09

**Figure:**
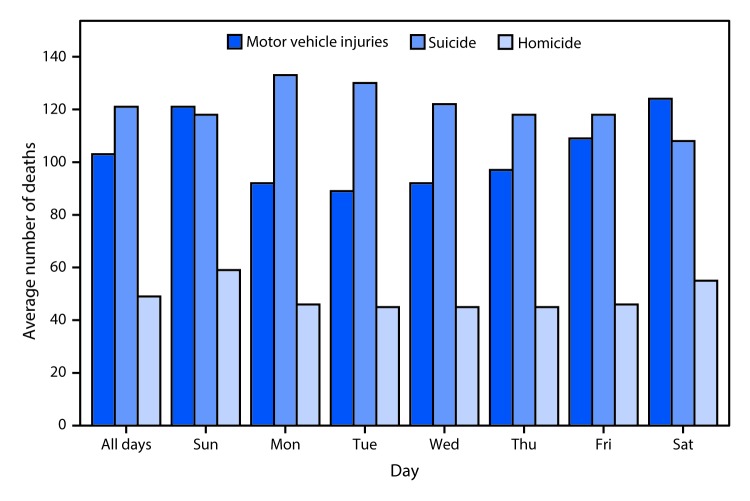
In 2015, an average of 103 motor vehicle injury deaths, 121 suicides, and 49 homicides occurred each day. Motor vehicle injury deaths were more likely to occur on Saturdays and Sundays and least likely to occur on Tuesdays. The highest number of suicides occurred on Mondays and Tuesdays and the lowest on Saturdays. Homicides peaked on Sundays, followed by Saturdays; homicides were less likely to occur on weekdays.

**Source:** National Vital Statistics System. Mortality public use data file, 2015. https://www.cdc.gov/nchs/data_access/vitalstatsonline.htm.

For more information on this topic, CDC recommends the following link: https://www.cdc.gov/injury.

